# Moderate High-Pressure Superdormancy in *Bacillus* Spores: Properties of Superdormant Spores and Proteins Potentially Influencing Moderate High-Pressure Germination

**DOI:** 10.1128/aem.02406-21

**Published:** 2022-02-22

**Authors:** Alessia I. Delbrück, Yvette Tritten, Paolo Nanni, Rosa Heydenreich, Alexander Mathys

**Affiliations:** a Sustainable Food Processing Laboratory, Institute of Food, Nutrition and Health, Department of Health Science and Technology, ETH Zurich, Zurich, Switzerland; b Functional Genomics Center Zurich, ETH Zurich and University of Zurich, Zurich, Switzerland; The Pennsylvania State University

**Keywords:** *Bacillus*, spore, superdormant, high-pressure, germination

## Abstract

Resistant bacterial spores are a major concern in industrial decontamination processes. An approach known as pressure-mediated germination-inactivation strategy aims to artificially germinate spores by isostatic pressure to mitigate their resistance to inactivation processes. The successful implementation of such a germination-inactivation strategy relies on the germination of all spores. However, germination is heterogeneous, with some “superdormant” spores germinating extremely slowly or not at all. The present study investigated potential underlying reasons for moderate high-pressure (150 MPa; 37°C) superdormancy of Bacillus subtilis spores. The water and dipicolinic acid content of superdormant spores was compared with that of the initial dormant spore population. The results suggest that water and dipicolinic acid content are not major drivers of moderate high-pressure superdormancy. A proteomic analysis was used to identify proteins that were quantified at significantly different levels in superdormant spores. Subsequent validation of the germination capacity of deletion mutants revealed that the presence of protein YhcN is required for efficient moderate high-pressure germination and that proteins MinC, cse60, and SspK may also play a role, albeit a minor one.

**IMPORTANCE** Spore-forming bacteria are ubiquitous in nature and, as a consequence, inevitably enter the food chain or other processing environments. Their presence can lead to significant spoilage or safety-related issues. Intensive treatment is usually required to inactivate them; however, this treatment harms important product quality attributes. A pressure-mediated germination-inactivation approach can balance the need for effective spore inactivation and retention of sensitive ingredients. However, superdormant spores are the bottleneck preventing the successful and safe implementation of such a strategy. An in-depth understanding of moderate high-pressure germination and the underlying causes of superdormancy is necessary to advance the development of mild high pressure-based spore control technologies. The approach used in this work allowed the identification of proteins that have not yet been associated with reduced germination at moderate high pressure. This research paves the way for further studies on the germination and superdormancy mechanisms in spores, assisting the development of mild spore inactivation strategies.

## INTRODUCTION

Spores are formed by multiple *Bacillales* and *Clostridiales* members under unfavorable conditions. These dormant spores are metabolically inactive and highly resistant to various environmental stressors. This stress resistance is attributed to a variety of features, including a layered structure, consisting of a spore core, a low-permeability inner membrane, a cell wall, an additional peptidoglycan cortex, an outer membrane, and several proteinaceous coat layers. Spore core DNA is protected by small acid-soluble proteins (SASPs), and the water content is minimized through various processes, including accumulation of dipicolinic acid (DPA) chelated with Ca^2+^ (1). Spores are ubiquitous in nature, and several pathogenic and spoilage spore-forming organisms are associated with human and foodborne diseases, food waste, and spoilage. One species, Bacillus anthracis, can even be used as a potential bioweapon, which happened in the United States in 2001. Accordingly, spore inactivation is of high relevance in a multitude of industries and contexts. Unfortunately, highly resistant spores pose a challenge in inactivation processes (2, 3). Intensive heat treatment is commonly applied to inactivate dormant spores; however, this treatment is associated with a significant loss in important quality attributes, such as nutritional value, color, or taste in the food sector or heat-labile active ingredients in the pharmaceutical and biotechnological sectors (3, 4). The scientific and industrial community therefore require cost-effective strategies that mildly inactivate spores to retain important quality attributes. One of the strategies under investigation is termed “germination-inactivation” or “germinate-to-eradicate” strategy. It aims to first germinate the spores so that they lose their resistance, after which the germinated spores can be easily inactivated using mild processes, such as mild heat or pressure treatment (5, 6). Different stimuli can trigger germination, including nutrients; surfactants, such as dodecylamine; external Ca^2+^-DPA; or isostatic high pressure (HP). From an industrial perspective, HP is of particular interest, as it does not require additional ingredients or chemicals, products are treated homogeneously, consumer acceptance is high, and the technology can be used to trigger germination as well as simultaneously inactivate germinated spores and other vegetative cells (7, 8). It has been shown that different germination pathways exist depending on the pressure and temperature level. Moderate HP (mHP; 50 to 300 MPa; 30 to 50°C) triggers germination through the germinant receptors (GRs) in a pathway similar to nutrient germination, whereas very HP (vHP; 400 to 600 MPa; <60°C) triggers germination independent of the GRs by directly releasing the Ca^2+^-DPA (9–13). This study focused on mHP-mediated germination. mHP activates GRs in the inner membrane followed by Ca^2+^-DPA release through SpoVA proteins. Ca^2+^-DPA release is accompanied by partial water uptake and the activation of cortex degrading lytic enzymes (CLEs); this process leads to further water uptake and core swelling, which are essential for the loss of most of the resistance. SASPs protecting the core DNA are also degraded following Ca^2+^-DPA release (8, 13, 14).

Applications that could profit from the successful implementation of the mHP-mediated germination-inactivation approach include the following: (i) the decontamination of various food products, such as ready-to-eat meals, juices, beverages, meat, seafood, and baby food, while meeting consumer demands for fresh and nutritious food. Eradication of spores would allow for shelf-stable storage of a multitude of products that currently need cooling to avoid spore outgrowth, which is not only a logistical challenge but also an environmental burden (13). (ii) A second application is the sterilization of liquid or gel-like, heat-labile pharmaceuticals that are not suitable for sterile filtration while maintaining the activity of sensitive compounds (3). (iii) A third application is the sterilization of bones, cartilage, and tendon *ex vivo* while leaving the mechanical properties of the tissues unimpaired, thus allowing reimplantation of the resected tissue explants (15).

However, the successful application of a germination-inactivation strategy is hampered by heterogeneous spore germination. Heterogeneity in germination velocity is observed for all germination triggers among species, but also within an isogenic population (2, 6, 7, 16–19). Of special concern are superdormant (SD) spores, which do not germinate at all or only extremely slowly compared with the rest of the (isogenic) population (6). Superdormancy is a relative term for spores within a population that are the least responsive or most resistant to a specific germination trigger. Hence, prevalence depends on the selection pressure, the type of germination trigger, and germination treatment conditions (e.g., pressure, temperature, and time) (20). SD spores may survive the mild inactivation step and germinate later during storage, potentially causing spoilage and/or disease.

Spores that are SD to nutrients have been characterized extensively (21–26). Given the similarity in the germination pathways between nutrient- and mHP-mediated germination, it is likely that commonalities exist in the characteristics of SD spores (14). The prevalence of nutrient SD spores is reportedly influenced by the following factors: (i) nutrient concentration and time of exposure; (ii) heat activation, whereas a sublethal heat treatment prior to nutrient germination decreases the SD population; and iii) GR levels, whereas lower levels of GRs are associated with superdormancy. These findings likely hold true for most *Bacillus* species. An observed loss of superdormancy upon extended storage at 4°C gave rise to the hypothesis that superdormancy may be caused by two factors, as follows: one permanent cause (probably GR levels) and one transient cause (potentially spore activation status) (21, 23, 25–27). However, evidence suggests that other factors may also be involved in nutrient superdormancy. Chen et al. (26), for example, proposed decreased levels of GerD as another contributing factor to nutrient superdormancy. GerD is known to be critical for germinosome assembly and hence rapid GR-dependent germination (28, 29). Furthermore, Ghosh et al. (23) observed that nutrient SD spores have a lower core water content and increased temperature requirements for heat activation. One cause of nutrient superdormancy that appears to be ruled out is genetic change, for example, in a critical part of the germination machinery (24).

The factors that influence mHP-mediated germination are partially similar to those of nutrient germination. Elevated GR levels have been shown to increase mHP germination at 150 MPa (11). How these GRs are activated by mHP is unclear, but it seems likely that conformational changes are involved. Increasing pressure dwell times also reduced SD spores (7, 20). In contrast, heat activation does not increase mHP germination (20, 30). It is only recently that mHP SD spores have been isolated and partially characterized (7, 20). It was shown that structural differences, such as cortex thickness or general spore size, do not seem to cause superdormancy. Similar to nutrient superdormancy, it was found that mHP superdormancy does not seem to be caused by genetic alterations. While genetic changes seem to be ruled out as cause of the SD phenotype, stochastic variations in gene expression may influence the phenotype.

The aim of this study was to gain further understanding of mHP germination and potential underlying causes of spore mHP superdormancy. As the low core water content likely causes the minimal metabolic activity and hence dormancy in spores (31), the spore core water content and DPA content were compared between SD spores and the initial dormant spore population. To identify proteins with significantly differential expression in superdormant spores, a quantitative comparison of proteins in the initial dormant spore population and SD spores was conducted. The role of selected proteins in mHP germination was further validated by investigating the mHP germination capacity of deletion mutants lacking these specific proteins.

## RESULTS

### Core water content in dormant and SD spores.

**FIG 1:**
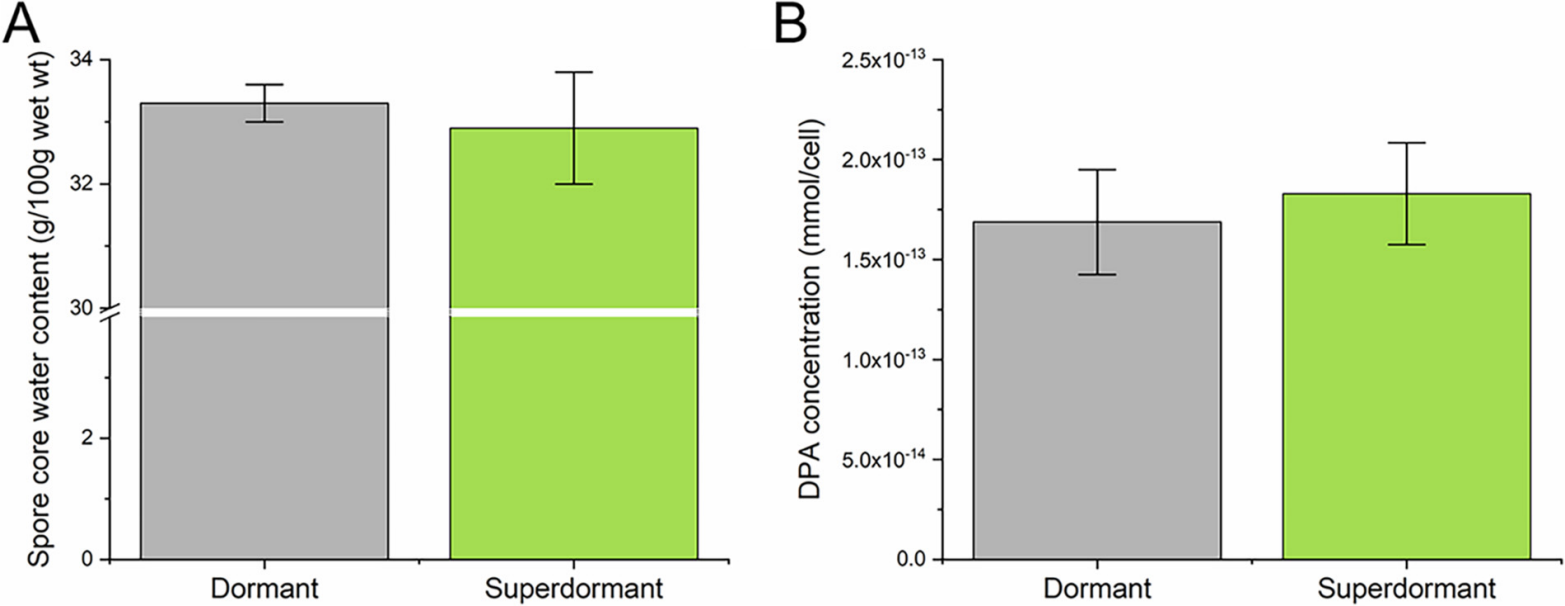
Comparison of core water content (g/100 g wet weight) (A) and DPA content (mmol/cell) (B) in the initial dormant Bacillus subtilis spore population (dormant) and high-pressure superdormant (SD) spores. SD spores were isolated after high-pressure treatment at 150 MPa and 37°C for 6 min in 50 mM ACES (pH 7). The core water content was determined using buoyant density gradient centrifugation and the DPA content using a terbium DPA fluorescence assay as described in the Materials and Methods. Both core water content and DPA concentration were similar between the initial dormant spore population and SD spores. The error bars are presented as standard deviations of *n* ≥ 3.

To investigate the influence of water content on superdormancy, the core water content was determined using buoyant density gradient centrifugation on Nycodenz gradients and compared between the initial dormant spore population (referred to as “dormant population” in the following) and SD spores isolated after HP treatment (150 MPa; 37°C; 6 min). The purity of isolated SD spores was 90% ± 6%. Impurities of germinated spores in these samples were separated from the SD spores during centrifugation on Nycodenz gradients due to strong differences in density. Coat removal of dormant and SD spores prior to the analysis was successful; the spores germinated upon addition of lysozyme and turned phase dark under a phase-contrast microscope (data not shown). The determined spore core wet densities were 1.375 ± 0.001 g/mL (*n* = 3) and 1.376 ± 0.002 g/mL (*n* = 5) for dormant and SD spores, respectively. The core water content in dormant and SD spores was very similar, with 33.3 ± 0.3 g/100 g wet weight (*n* = 3) and 32.9 ± 0.9 g/100 g wet weight (*n* = 5), respectively (Fig. 1A).

### DPA content in dormant and SD spores.

As a potential influencing factor of superdormancy, DPA content was compared between dormant and SD spores using a terbium DPA fluorescence assay. SD spores were isolated after HP treatment (150 MPa; 37°C; 6 min). The purity of the SD spores was 91% ± 5% and that of dormant spores was ≥ 99%. The purity of the samples was normalized to 100% (super)dormant spores, based on the assumption that germinated spores previously released all their DPA and hence released none in the autoclaving step. Dormant and SD spores had a similar DPA content, with 16.9 × 10^−14^ ± 2.6 × 10^−14 ^mmol/cell (*n* = 3) and 18.3 × 10^−14^ ± 2.5 × 10^−14 ^mmol/cell (*n* = 3), respectively (Fig. 1B).

### Proteome analysis and selection of proteins of interest.

**FIG 2:**
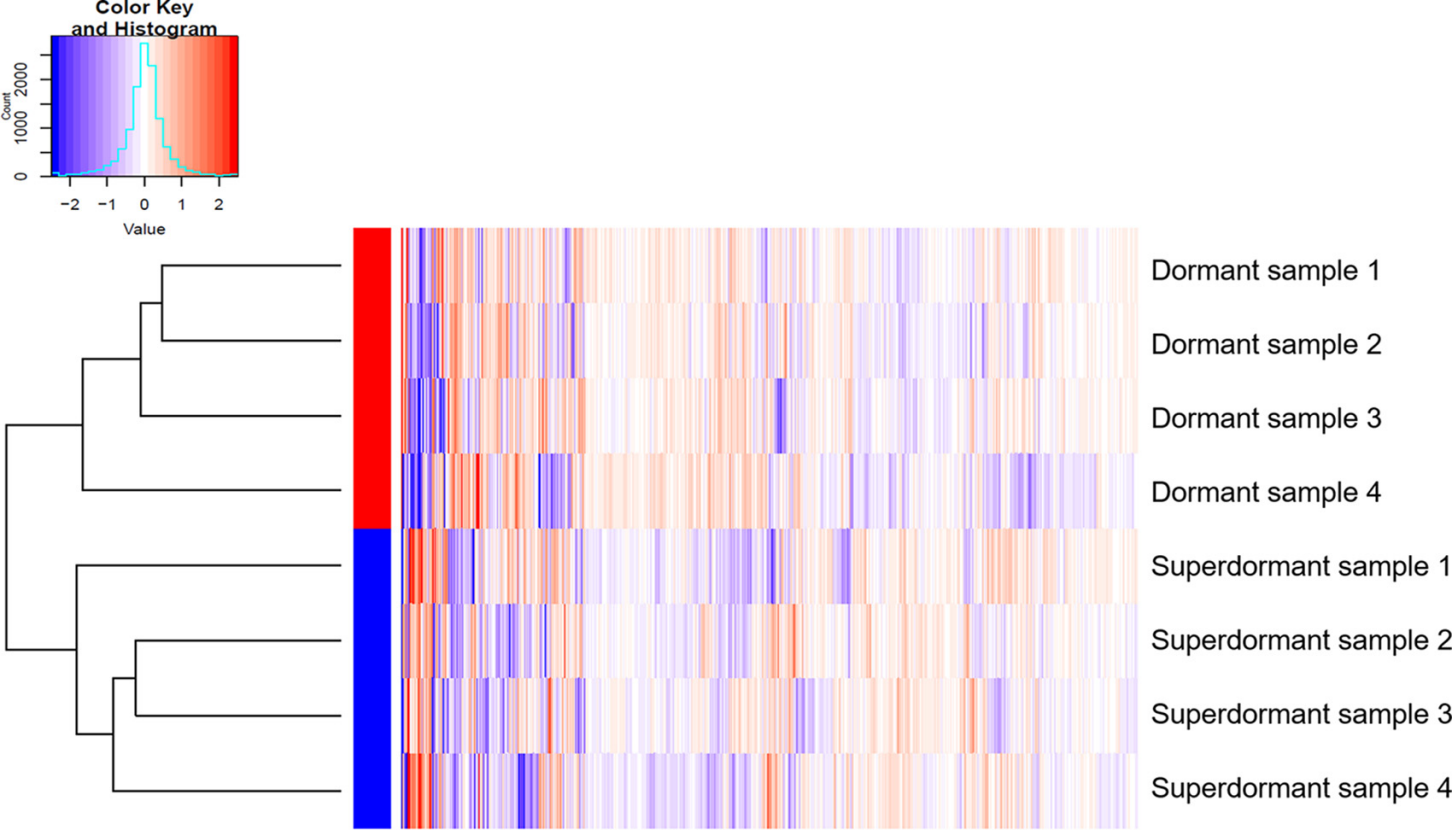
Heatmap of normalized data of proteomic analysis of the initial dormant Bacillus subtilis spore population (here called “dormant”) and the superdormant spores isolated after a high-pressure treatment at 150 MPa and 37°C for 4 min. Each single vertical line represents a protein. The color of the line indicates whether this protein is more abundant (red), less abundant (blue), or similarly expressed (white) when comparing the various samples. The dendrogram at the left indicates the similarities between the single samples and thereby clusters the samples according to how similar their overall protein expression is. The variance in protein expression within the replicates of dormant or superdormant spores was smaller than the variance observed between the two sample types.

**FIG 3:**
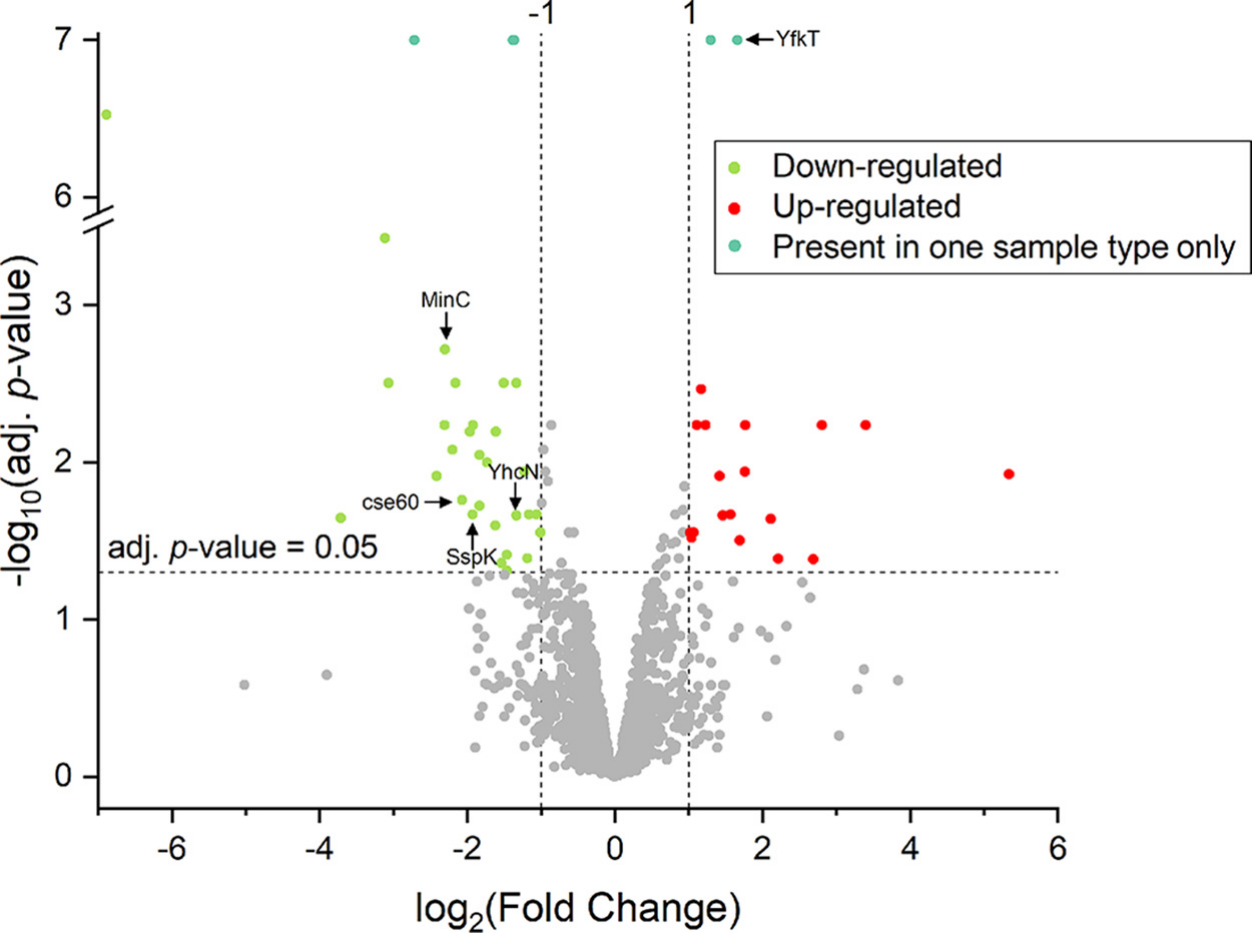
Volcano plot visualizing the statistical significance and magnitude of difference in protein quantity between the initial dormant Bacillus subtilis spore population (“reference”) and the high-pressure superdormant spores (“condition”) isolated after a high-pressure treatment at 150 MPa and 37°C for 4 min. The red and green dots represent proteins that are significantly lower or higher in abundance (adjusted *P* < 0.05) with a log_2_ fold change [log_2_(FC)] of >|1|. The turquoise dots represent proteins that were present only in one sample type. A full list of the proteins that were significantly differentially expressed can be found in the Table S2 in the supplemental material.

Label-free protein quantification was conducted with eight samples, consisting of four dormant spore samples (“reference”) and four SD spore samples (“condition”). SD spores were isolated after HP treatment (150 MPa; 37°C; 4 min) with a purity of 94% ± 3%. Approximately 1,500 proteins were quantified with >2 peptides, <4 missing values (see Table S1 in the supplemental material), and a false discovery rate (FDR) of 2.1%. Protein levels in dormant and SD spores were examined for differential expression using a null hypothesis significant test. Significantly differentially expressed proteins were defined based on an adjusted P value of <0.05, and a log_2_(fold change [FC]) of ≥|1|, whereas a log_2_(FC) of <0 means that this protein is present in smaller amounts in the SD sample than that in the dormant sample; a log_2_(FC) of >0 means that this protein is present in larger amounts in the SD sample. From Fig. 2, it is visible that the natural variance in protein expression within the replicates of dormant and SD spores was smaller than the variance observed between the two sample types. SD spores showed a significantly different protein expression compared with the dormant spores, more so than the natural variation between the replicates. Among the quantified proteins, 52 B. subtilis proteins were significantly differentially expressed between dormant and SD spores or only identified in one of the two sample types (Fig. 3; see Table S2 in the supplemental material). It is worth noting that the SD samples had some impurities (approximately 6%) of germinated spores. However, the purity of the SD spore samples did not correlate with detected protein quantities. It can therefore be excluded that small impurities coming from germinated spores influenced the results in a significant way. The 52 identified proteins were screened for their putative function in the B. subtilis strain 168 database from the UniProt consortium. Table 1 shows a list of proteins selected for further investigation based on the following criteria: (i) significantly different expression in dormant and SD spores, (ii) reportedly associated with sporulation or germination, (iii) direct availability of a mutant strain lacking the gene coding for this protein for further validation, and (iv) successful sporulation in parallel to the wild type.

**TABLE 1:** Selection of proteins for further analysis

Protein name	Accession no.	Description in UniProt	Gene	log_2_(FC)^*a*^	Adj. *P* value	Protein quantification
MINC_BACSU	Q01463	Septum site-determining protein MinC	*minC*	−2.3	0.002	↓Approx; 5× less in SD spores
CSE60_BACSU	P94496	Sporulation protein cse60	*cse60* (synonym *yteV*)	−2.1	0.017	↓Approx; 4× less in SD spores
SSPK_BACSU	Q7WY75	Small, acid-soluble spore protein K	*sspK*	−1.9	0.022	↓Approx; 4× less in SD spores
YHCN_BACSU	P54598	Probable spore germination lipoprotein YhcN	*yhcN*	−1.3	0.022	↓Approx; 2.5× less in SD spores
YFKT_BACSU	O34573	Putative spore germination protein YfkT	*yfkT*	NA^*b*^	NA	In SD spores only

a log_2_(FC), log_2_ fold change; indicates whether a certain protein was more (log_2_(FC) > 0) or less (log_2_(FC) < 0) abundant in the superdormant (SD) than the dormant (D) Bacillus subtilis spore sample.

b NA, not applicable.

### Validation of the influence of selected proteins in mHP germination.

**FIG 4:**
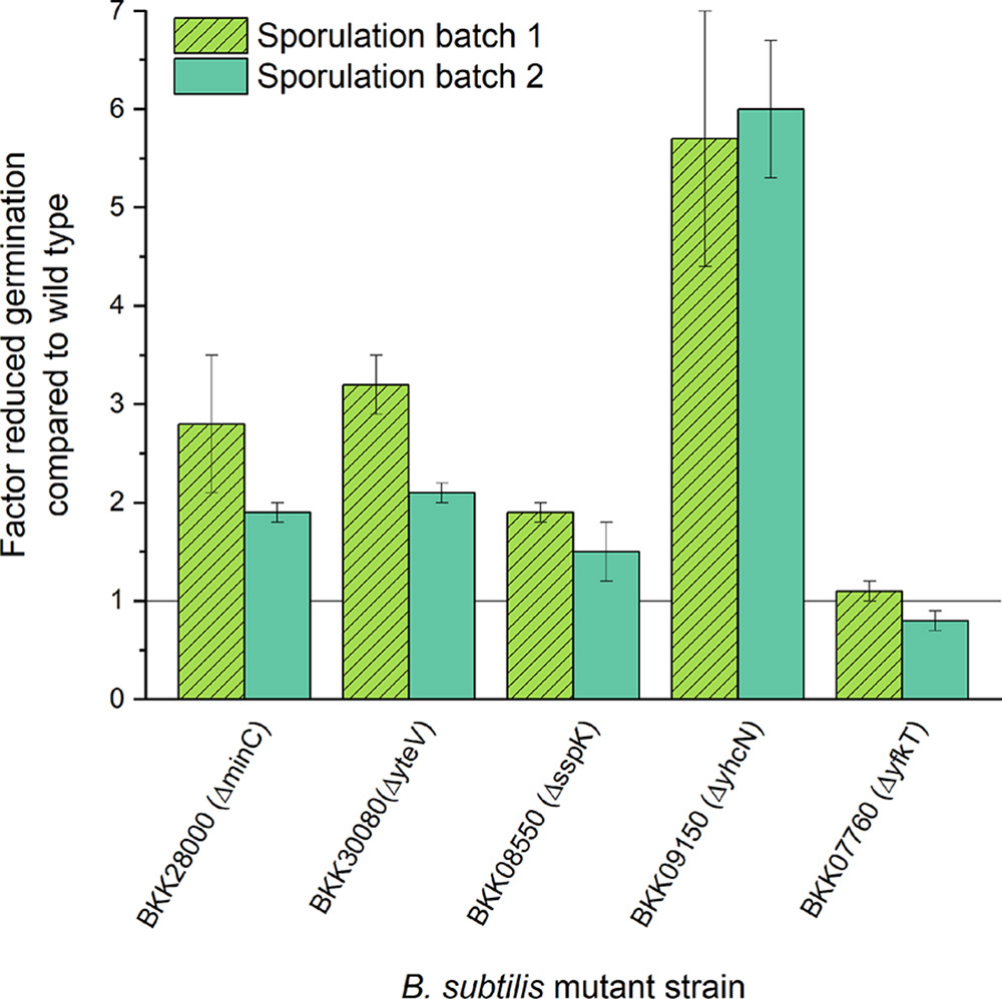
Germination capacity of Bacillus subtilis mutant strains lacking the genes for selected proteins compared with that of the otherwise isogenic wild-type 168 strain. Wild-type and mutant strains were sporulated twice in parallel in two independent sporulation batches. Spores of a mutant and wild-type strain were high-pressure-treated simultaneously in triplicate at 150 MPa and 37°C for 3 min in 50 mM ACES (pH 7), and the percentages of germinated and superdormant spores were determined using flow cytometry. The factor by which germination is slowed down in mutant strains compared with the wild-type strain was calculated by comparing the % of superdormant spores left after high-pressure treatment. Error bars represent standard deviations of three independent experimental replicates (*n* = 3) for each sporulation batch. The horizontal line indicates the germination capacity of the wild type. All mutant strains, except for *yfkT*, showed a reduced germination capacity. *yteV* is synonym for *cse60*.

To independently validate the role of potential proteins of interest in mHP germination, the germination capacity of B. subtilis deletion mutants lacking the specific proteins was compared with that of the otherwise isogenic wild-type strain B. subtilis 168 (Fig. 4). Mutant and wild-type spores were HP treated at 150 MPa and 37°C for 3 min, and the remaining SD spores were quantified using flow cytometry (FCM). Except for the mutant strain BKK07760 (*ΔyfkT*), which showed a germination capacity similar to that of the wild type, all other mutant strains showed reduced germination, represented by an increased percentage of SD spores after the treatment. In particular, the mutant strain lacking the YhcN protein showed strongly reduced germination with an approximately 6-fold reduced germination. After 3 min of HP treatment, 61.8% ± 3.2% of spores of the *yhcN* mutant strain remained dormant in one sporulation batch and approximately 40% germinated. This finding is in contrast to the wild type, where after the same pressure treatment, only 10.3% ± 0.7% of spores remained dormant and approximately 90% germinated. The other sporulation batch showed a similarly reduced germination with 28.4% ± 5.8% SD spores in the mutant strain compared with 5.0% ± 0.2% SD spores in the wild-type strain.

The factor by which germination was reduced varied slightly between different sporulation batches. However, the observation that all mutant strains except BKK07760 (*ΔyfkT*) showed reduced germination compared with the wild-type strain holds true for all experimental replicates and sporulation batches that were prepared independently (Fig. 4).

## DISCUSSION

### Core water content and DPA content of dormant and SD spores.

Low core water content is a major reason for the minimal activity of spores and significantly contributes to dormancy, likely by reducing molecular mobility in the core. Different factors have been reported to influence the water content in dormant spores, including the availability of different types of metal cations, sporulation temperature, and DPA content (32–34). DPA binds and chelates Ca^2+^ ions and plays a key role in the dehydration and mineralization of the core (32, 35).

The determined core water content of approximately 33% of wet weight for both dormant and SD spores was within the literature reported range of 25% to 45% during dormancy (8). In addition, the determined DPA content of around 10^−13 ^mmol/cell is similar to that previously reported (36–38). No remarkable differences in core water content or DPA content were observed between the initial dormant spore population and SD spores. Based on the above-described interdependencies of water and DPA content, it seems consistent that neither of the two parameters varied significantly between dormant and SD spores.

Considering the reported similarity between mHP and nutrient germination (14), it is interesting to compare these findings to similar investigations conducted on B. subtilis nutrient SD spores (23, 24). Ghosh et al. (23) found that, contrary to our findings on mHP SD spores, the nutrient SD spores had a lower core water content than the original dormant spore population. Interestingly, the DPA levels were essentially identical between dormant and nutrient SD spores (24). Since the same amount of DPA was associated with different amounts of water in the two types of spores, Ghosh et al. (23) suggested that there may be notable differences in the environment of DPA in dormant and SD spores. To examine this possibility, the authors conducted laser tweezers Raman spectroscopy on individual spores. They found that the environment of DPA in the SD spore core differed from that of the initial dormant spores. However, what this means in molecular terms is unclear. It is possible that lower core water content influences nutrient but not mHP superdormancy. However, when comparing the properties of SD spores, it is necessary to consider the isolation conditions, as superdormancy is a relative term (14, 20). Ghosh et al. (23) isolated nutrient SD spores by germinating spores with a saturating concentration of 10 mM l-valine for 2 h at 37°C, removing the germinated spores, and repeating. Hence, they applied two selection cycles for SD spores, unlike this study where SD spores were isolated after one pressure cycle. In this study, it would not have been possible to isolate SD spores after two pressure cycles as the proportion of the remaining SD spores would have been too small to isolate sufficient amounts for further analysis. Therefore, the selection for superdormancy was potentially more stringently chosen by Ghosh et al. (23). Accordingly, it is possible that SD spores isolated after two pressure cycles or after longer pressure dwell times would also show a significantly different spore water content compared with the initial dormant spore population. On the other hand, the isolation procedure applied by Ghosh et al. (23) yielded 1% to 3% nutrient SD spores. This is similar to our selection process, which yielded approximately 3% SD spores (3.2% ± 0.5%) for this experiment. Therefore, in both cases, the 1% to 3% slowest germinators within a population were isolated and characterized, which could be interpreted as a similarly stringent definition of superdormancy. In conclusion, it cannot be excluded that SD spores obtained from a more stringent isolation protocol may also have lower water content. However, it seems clear that the water content and DPA content do not seem to be the dominant and driving causes of mHP superdormancy, at least not when characterizing the 3% slowest germinators in a population.

### Proteome analysis, identification, and validation of proteins potentially involved in mHP germination.

The proteome analysis of dormant and SD spores identified approximately 1,500 proteins in B. subtilis PS533. This finding is comparable to or even slightly more than other reported B. subtilis spore proteome analyses (39, 40). Fifty-two proteins were found to be significantly differentially expressed. Surprisingly, GRs, which are triggered by mHP and in turn start the germination cascade, were not among the 52 proteins. GerAA, GerAC, GerBA, GerKA, and GerKC were detected; however, they did not significantly differ in levels between dormant and SD spores. This result was unexpected because the levels of GRs, especially GerA, are thought to influence mHP superdormancy (11, 41). There is evidence that GR levels alone cannot account for all the heterogeneity and hence superdormancy observed in mHP-mediated germination (14). However, it is difficult to explain why no significant difference in expression was found. Doona et al. (41) hypothesized that GerD or the D subunits of GRs may influence mHP-mediated spore germination. Unfortunately, the D subunits of GRs were not detected in our proteome analysis. GerD was detected and showed no significant differences in levels. Mutants lacking *gerD* have been shown to germinate slower with nutrients as well as with an mHP of 150 MPa (42). Researchers have investigated the effect of GerD on nutrient superdormancy, and Ghosh et al. (21) found identical levels of GerD proteins in dormant and nutrient SD spores using Western blot analysis. Chen et al. (26) found that GerD was significantly less abundant in nutrient SD spores than that in dormant spores, as analyzed using multiple-reaction-monitoring liquid chromatography-mass spectrometry (LC-MS) assays. Clearly, further research is needed concerning the role of GerD in both nutrient- and mHP-mediated germination. Generally, as discussed above, the stringency with which SD spores are isolated may influence the outcome of such studies. The present study focused on 5 selected proteins among the 52 proteins that were significantly differentially expressed for further analysis. It is worth noting that the selection of proteins of interest from the proteome analysis is by no means exhaustive and served mainly to narrow down potential protein candidates of interest. There are likely other proteins involved in mHP germination and potentially superdormancy that were not identified through this approach because, for instance, their role in germination or sporulation has not yet been discovered and/or deposited in UniProt or simply because this first screening did not show enough difference in expression between dormant and SD spores to meet the criteria for a significantly different expression defined in this approach. Some more mutant strains were tested; however, they were difficult to sporulate and were therefore excluded from further analysis (data not shown). The role of the 5 selected proteins in mHP germination was validated by mutant strains lacking the genes coding for these proteins. Except for the Δ*yfkT* mutant, all mutant strains had a reduced germination capacity compared with the wild type suggesting that these proteins indeed seem to play a role in mHP germination and potentially superdormancy. However, it is worth highlighting that reduced protein levels in SD spores seen in the proteomic analysis may correlate with them being superdormant; however, they do not necessarily present a causality for superdormancy. The validation with the deletion mutants gave further evidence that certain proteins, particularly YhcN, indeed seem to be significantly important for effective mHP germination. While this work identified proteins that appear to influence mHP germination, further evidence is needed to determine whether they are important for superdormancy in wild-type spores.

In the following these proteins and their putative function are discussed briefly.

### (i) MinC.

MinC is a part of a protein complex called the Min system which ensures symmetrical division of multiplying vegetative cells. Interestingly, the Min system of B. subtilis also seems to play a role during sporulation, a process that begins with asymmetrical cell division. It is not understood how the inhibitory effect of the Min system on polar division is overcome during sporulation (43, 44). However, it was shown that in the majority of cells lacking MinCD, the septum position moves closer to the cell pole compared with normal septation at the beginning of sporulation. These *minCD* mutant spores tend to be slightly longer (45). The length of spores, however, is similar in mHP SD spores and in the initial dormant spore population (20). Therefore, abnormal cell length is an unlikely explanation for the reduced germination.

In the proteome analysis, the MinC protein was less abundant in the SD spores than in the initial dormant population, and deletion of the *minC* gene led to decreased germination, suggesting that lower levels of MinC may negatively impact germination. However, it is difficult to hypothesize what role it may play in germination or how a more polar septum placement during sporulation may influence the final spore and eventually germination properties.

### (ii) YhcN.

YhcN is a predicted lipoprotein that has been detected in the inner spore membrane as well as in the outer spore layers (46–48). Interestingly, similar to this study on mHP germination, Johnson and Moir (49) showed that mutants lacking YhcN showed a reduced rate of germination in l-alanine. Although the function of this protein is not yet understood, recent research has shed some light on its potential role in germination. YhcN contains a common domain with another protein called YlaJ. It was shown that SleB activity was compromised in spores that lacked both *ylaJ* and *yhcN* genes in a Δ*cwlJ* mutant background. This finding suggests that YhcN and YlaJ contribute, directly or indirectly, to effective SleB function during germination. Additionally, Johnson and Moir (49) observed not only a delay in the release of DPA and OD fall during l-alanine germination but also a delay in the loss of heat resistance in *yhcN* mutants. As loss of heat resistance is considered an early event in germination (50), the authors suggested that the role of YhcN may not be limited to its effect on SleB function but may also influence germination events earlier than cortex hydrolysis. This accords with the finding that heterogeneity in both nutrient and mHP germination is attributed mainly to variability in T_lag_, the time between trigger application and initiation of rapid Ca^2+^-DPA release, and hence events prior to cortex hydrolysis (19). Johnson and Moir (49) further investigated whether increasing the permeability of germinants could overcome the defect in *yhcN* mutant germination with l-alanine. As this did not succeed, the authors concluded that the defect in *yhcN* mutant spores was not due to a reduction in the coat permeability of the spores to germinants. The fact that the germinant-independent mHP germination also seems to be affected negatively in *yhcN* mutant spores is consistent with this conclusion. As YhcN was less abundant in SD spores and germination was reduced in the absence of YhcN, it is suggested that the protein has a positive effect on germination.

### (iii) cse60.

Slower mHP germination was observed in the *cse60* deletion mutants. More than 20 years ago, Henriques et al. (51) reported the characterization of a new transcription unit *cse60* (synonym for *yteV*). However, the function remains unknown to date, and further research is needed to verify whether and how this protein plays a relevant role in mHP germination.

### (iv) SspK.

SASPs are proteins present in the spore core. SASPs are commonly classified into major (α, β, and γ) and minor SASPs (52). SspK is a minor SASP and is expressed in the forespore compartment of sporulating cells (52–55). Cabrera-Hernandez et al. (52) found that there was no difference in spore germination triggering and outgrowth with the nutrients l-alanine and l-tryptophan between an *sspK* mutant and wild-type strains, as established by monitoring the optical density at 600 nm (OD_600_). This result is in contrast to our findings, where mutant strains lacking SspK germinated slightly slower than the wild-type strain under pressure. However, the observed reduction was rather minor. This difference in observation could be attributed to different methods used to monitor germination, or possibly, this protein may influence mHP germination but not l-alanine/l-tryptophan-triggered germination. Further insights into this protein and its potential role in dormant spores are needed to draw further conclusions.

### (v) YfkT.

This protein was the only protein in which mutant validation did not show reduced germination. Instead, the Δ*yfkT* mutant showed a similar germination capacity to the wild type, suggesting that this protein is not of particular relevance in mHP germination. This protein is encoded by the *yfkQRT* operon, a homolog of the *gerA* operon that encodes GR GerA (56). Similar to our findings, whereas deletion of *yfkT* did not influence mHP germination, Paidhungat and Setlow (56) showed that deletion of the *yfkQRT* operon had no effect on the ability of spores to germinate in response to nutrients. In the proteomics analysis, this protein was not identified in the dormant sample but was identified in the SD sample with very low intensity. A potential explanation for the deviation of the proteomic analysis to its independent validation with mutant strains may be that the protein signals were at the limit of detection. The real difference may not be its presence/absence but rather a tiny fold change, which is not enough to significantly influence mHP germination.

### Conclusions.

The present study sheds more light on mHP germination and the still poorly understood underlying cause of mHP superdormancy. Previous studies have suggested that structural causes, such as cortex thickness or general spore size, are unlikely causes of superdormancy, as are genetic alterations. Furthermore, it was suggested that GR levels alone are unlikely to explain superdormancy (14, 20). This study provides evidence that water content and DPA content are also unlikely to be major drivers of superdormancy. Based on the principle of exclusion, it seems reasonable to hypothesize that variations in the expression of certain proteins may be responsible for heterogeneous spore germination and mHP superdormancy. This study used a proteomic approach to narrow down potential protein candidates for further investigation. It was found that the proteins MinC, cse60, YhcN, and SspK all seem to positively influence mHP germination, as their absence led to a reduction in the germination capacity of deletion mutants, especially the protein YhcN. Further research is needed to determine their putative role in mHP germination and superdormancy. Of particular interest may be further research into the protein YhcN and its role in mHP germination and potentially superdormancy. The reasons include the following: (i) the absence of this protein led to a 6-fold reduction in germination rate under mHP, (ii) homologs of YhcN are widespread in *Bacilli* and *Clostridia*, and (iii) the loss of *yhcN* has recently also been associated with a reduced rate of spore germination with l-alanine (49). There are likely other proteins that also contribute to mHP germination and superdormancy, and the evidence seems to point toward a complex interplay of several factors influencing mHP superdormancy. Surprisingly, this study did not identify significantly different levels of GRs in dormant and SD spores. A follow-up regarding the precise role of GRs in mHP germination and superdormancy would be valuable. While further research is needed, this study brought us a step forward in excluding water and DPA content as major drivers of mHP superdormancy and identifying proteins that appear to play a role in mHP germination. This information is a starting point for additional work in this area.

## MATERIALS AND METHODS

### Bacillus subtilis strains and spore preparation.

For the proteome analysis as well as water and DPA content measurement, B. subtilis strain PS533 was used; it is an isogenic mutant of strain PS832, which in turn is a laboratory derivative of the widely used strain 168. PS533 carries the kanamycin resistance-conferring plasmid pUB110 (57). For validation of the proteomics analysis, mutant strains lacking genes encoding selected proteins were obtained from the Bacillus Genetic Stock Center (BGSC; Columbus, Ohio). The mutants included B. subtilis BKK28000 (*ΔminC::kan*), BKK30080 (*ΔyteV::kan*), BKK08550 (*ΔsspK::kan*), BKK09150 (*ΔyhcN::kan*), and BKK07760 (*ΔyfkT::kan*) (57). The parental strain 168 (called 1A1 at the BGSC) of these mutants, referred to as the wild type, was also obtained from the BGSC (Table 2).

**TABLE 2:** Bacillus subtilis strains

B. subtilis strain	Genotype (phenotype)	Reference
PS533	Carries plasmid pUB110 (Km^r^)	65
168	Wild type	66
Derivatives of strain 168		
BKK28000	*ΔminC::kan*	57
BKK30080	*ΔyteV::kan*	57
BKK08550	*ΔsspK::kan*	57
BKK09150	*ΔyhcN::kan*	57
BKK07760	*ΔyfkT::kan*	57

Overnight cultures of B. subtilis in tryptic soy broth with 10 μg/ml kanamycin for strain PS533, 5 μg/mL kanamycin for all other mutant strains, and no antibiotic for strain 168 were streaked onto Difco sporulation medium (DSM; pH 7.6). The plates were incubated for 3 to 5 days at 37°C. Mutant strains and wild-type strains were incubated in parallel to eliminate potential batch differences due to differing sporulation conditions. The sporulation progress was monitored every day using a phase-contrast microscope (DM6; Leica Microsystems, Wetzlar, Germany), and spores were harvested when the phase-bright population reached >95%. Harvested spores were washed at least four times on the day of harvest and twice daily for the consecutive week at 6,000 × *g* for 10 min with sterile Milli-Q water. The spores used in this study were ≥98% phase-bright dormant spores. The absence of unreleased spores, germinated spores, and cell debris was determined using phase-contrast microscopy and flow cytometry. Spores of strain 168 and its mutant strains were additionally purified using centrifugation through a high-density medium, as explained in detail in “Isolation of superdormant spores.” Spores were stored in the dark at 4°C in sterile Milli-Q water and washed every 2 weeks.

### HP-triggered spore germination.

Spores were HP treated at 150 MPa and 37°C in 50 mM 0.1-μm-filtered *N*-(2-acetamido)-2-aminoethanesulfonic acid (ACES; Thermo Fisher, Kandel, Germany) buffer solution at pH 7.0 for 3 to 6 min. A dual-vessel high-pressure unit (modified model U111; Unipress, Warsaw, Poland) was used for sample treatment, as described elsewhere (7, 20). B. subtilis PS533 spores that were further processed to isolate SD spores were treated in 1.5-mL cryotube vials sealed with a sealing tube (Nunc CryoFlex tubing; Nunc A/S, Roskilde, Denmark) at a concentration of approximately 10^10^ spores/mL. For the experiments where the germination of B. subtilis 168 wild-type spores were to be compared with that of deletion mutants, samples were treated in smaller polyethylene pouches of approximately 200 μL (heat shrink tubes; Hyperion, Hong Kong) at a concentration of approximately 5 × 10^8^ spores/mL to allow parallel treatment of wild-type spores and deletion mutants. Representative pressure/temperature/time profiles of the HP treatments can be found in the supplemental material (Fig. S1). An untreated control was prepared for each experiment and strain and stored on ice until further analysis. HP treatments were performed at least in triplicate on three different days. The results are expressed as mean ± standard deviation.

### Isolation of superdormant spores.

In this study, SD spores were defined as the spore fraction that did not germinate under pressure after treatment at 150 MPa and 37°C for 4 or 6 min. Approximately 10^10^ spores in 1.4 mL 50 mM ACES buffer (pH 7) were HP treated as described in “HP-triggered spore germination.” The percentage of germinated and SD fractions was determined using flow cytometry, as described in “Determination of (super)dormant and germinated spore fraction.” HP-treated samples were incubated at 37°C for 40 min to allow uptake of water by germinated spores. This process increased the density difference between the germinated and SD spores. The SD spores were then isolated using buoyant density centrifugation, as described previously (20). Briefly, HP-treated spores were pelleted using centrifugation at 12,000 × *g* and 4°C (Micro Star 17R; VWR International GmbH, Dietikon, Switzerland) and resuspended in 100 μL 20% of the density gradient medium Nycodenz (Axis-Shield, Oslo, Norway). This solution was carefully layered on top of 1.8 mL 50% Nycodenz in an Eppendorf tube. Afterward, the tube was centrifuged for 45 min at 13,000 × *g* and 4°C. The SD spores formed pellets due to their high density, while the germinated spores floated on top, as they had a lower density due to the absorbed water. The top layer and supernatant were removed carefully, and the pellets were resuspended in sterile Milli-Q water. The SD spores were washed again in sterile Milli-Q water using centrifugation at 12,000 × *g* for 2 min to ensure the removal of all Nycodenz and stored on ice for experiments conducted on the same day of isolation (water content and DPA determination) or at −20°C if the experiment was conducted within a few days after the isolation (proteome analysis). SD spores were always stored and handled the same way as their initial dormant spore counterparts to which they were to be compared. The purity of isolated SD spores was determined using flow cytometry, as described in “Determination of (super)dormant and germinated spore fraction.”

It is worth noting that the germination capacity between spore batches can vary, even if the same sporulation conditions are applied. As stated above, SD spores were isolated after 4 to 6 min of pressure treatment. Typically, 3% to 7% remained dormant after such treatment times, meaning that this study describes the characteristics of the ∼5% slowest germinators in the spore population. SD spores were freshly isolated for each experimental replicate.

### Determination of (super)dormant and germinated spore fraction.

The prevalence of (super)dormant spores and the number of germinated spores was assessed using an FCM-based method optimized and thoroughly validated by Zhang et al. (7). In short, samples were diluted in 0.1-μm-filtered Milli-Q water to approximately 10^7^ spores/mL and stained with the nucleic acid stains SYTO16 (Molecular Probes, Leiden, Netherlands) and propidium iodide (PI; Molecular Probes) at a final concentration of 0.1 μM and 1.5 μM, respectively. SYTO16 is indicative of germinated cells, whereas PI is indicative of germinated cells with compromised membranes. (Super)dormant spores do not stain with PI or SYTO16. For the analysis, stained samples were loaded in an LSR Fortessa cytometer (BD Biosciences, Franklin Lakes, USA) and 15,000 events were acquired per sample. Controls included 0.1-μm-filtered Milli-Q water to monitor background signals and an untreated sample of the initial dormant spores. The latter control had two functions, as follows: (i) to ensure that the initial dormant spore population had a high purity and therefore gave no signal in the SYTO16 and PI channels and (ii) to help set the gate of (super)dormant spores. No compensation was necessary for the chosen settings. The acquired fcs. files were analyzed using FlowJo software V10.6.1 (FlowJo LLC, Oregon, USA) using pseudocolor density plots on a biexponential scale. The same gating strategy was applied for each sample, as follows: (i) gating for single cells, thereby excluding cell agglomerates or multiple cells measured simultaneously, and (ii) gating for the fluorescent signals SYTO16 versus PI to identify (super)dormant, germinated, and membrane-compromised spores.

FCM was used to determine the initial dormant spore purity before treatment, the fraction of SD and germinated spores after HP treatment, and the purity of isolated SD spores.

### Determination of core water content.

To determine whether a difference in water content may contribute to superdormancy, the spore core water content was determined and compared between SD spores and the initial dormant spore population. SD spores were isolated as described in “Isolation of superdormant spores,” after a pressure dwell time of 6 min at 150 MPa. The spores were decoated as described by Ghosh et al. (23) by incubating approximately 2 × 10^9^ spores in 1 mL of decoating solution containing 0.1 M NaOH, 0.1 M NaCl, 1% sodium dodecyl sulfate, and 0.1 M dithiothreitol in Milli-Q water for 2 h at 70°C. After incubation, the sample was washed 10× with Milli-Q water (6,000 × *g*, 2 min, 4°C) and resuspended in 100 μL of 20% Nycodenz. To ensure that this decoating step was successful, control samples were treated in a 0.85% NaCl solution with 25 μg/μL lysozyme (Sigma-Aldrich, Steinheim, Germany), incubated at 37°C for 20 min, and analyzed under a phase-contrast microscope. Lysozyme is a lytic enzyme that can degrade the spore cortex and trigger germination, visualizable under a phase-contrast microscope by spores that turn from phase bright to phase dark (58). However, lysozyme can only access the cortex and trigger germination once the coat is removed (59). Hence, the germination of spores by lysozyme is proof of successful decoating. The core water content was then determined indirectly via a previously established correlation with the core wet density (60). The core wet densities, in turn, were determined by the principle of buoyant density centrifugation in a discontinuous Nycodenz gradient (73% to 68% [wt/vol]). The decoated spores of unknown density were layered on top of Nycodenz layers of known densities and centrifuged to equilibrium for approximately 45 min at 4,800 × *g* and 4°C in a swing bucket rotor (centrifuge Z 366 K; Hermle Labortechnik GmbH, Wehingen, Germany). Equilibrium is reached once buoyancy equals the centrifugal force. Consequently, the spores formed a band at a gradient layer of equal density. It is worth knowing that the decoating solution removes much of the spore coat proteins and the outer membrane (23). This leaves the spore core and the cortex. The cortex, however, is permeable for Nycodenz, and therefore, the measured density is representative of the core only (60).

The core water content was then determined using equation 1, where *x* is the core water content in g per 100 g wet weight and *y* the core wet density in g/mL (23, 34, 60).

(1) $$x=\frac{\left(1.460-y\right)}{0.00254}$$

HP treatment, isolation of SD spores, and subsequent water content determination were repeated at least three times on different days. The results are expressed as the mean of the replicates ± standard deviation.

### Determination of spore DPA content.

The spore DPA content was determined using fluorescence spectrometry and compared between SD spores and the initial dormant spore population. SD spores were isolated as described in “Isolation of superdormant spores,” after a pressure dwell time of 6 min at 150 MPa. Approximately 2 mL of spores at a concentration of approximately 10^8^ cells/mL were washed twice with Milli-Q water at 12,000 × *g* and 4°C for 5 min to remove potentially present external DPA. The exact spore concentration was determined using a counting chamber (improved Neubauer hemocytometer; Paul Marienfeld GmbH & Co, Lauda-Königshofen, Germany). The spores were autoclaved at 121°C for approximately 30 min to release all the internal DPA. After being autoclaved, the samples were placed on ice and sterile filtered (0.1-μm filter) to separate the cells from the supernatant containing the DPA. DPA quantification was then performed using a terbium-DPA fluorescence assay, as described previously (38). The terbium cation (Tb^3+^) reacts with the dipicolinate anion (DPA^2−^) to form a chelate, [Tb(DPA)_3_]^3−^, which has a strong and distinctive fluorescence (61). A calibration standard curve was recorded using analytical-grade DPA (Sigma-Aldrich, Steinheim, Germany) in the range of 0 to 150 μM. The linearity of the calibration curve was ensured (R^2^ ≥ 99%). In a nontransparent 96-well plate (SPL Life Science, Gyeonggi-do, South Korea), 100 μL of sample and calibration standard were mixed with 100 μL of a 20 mM TbCl_3_ stock solution (Sigma-Aldrich) prepared in 50 mM Trizma buffer (pH 7.5; Sigma-Aldrich). Fluorescence was measured using a fluorescence spectrometer (Plate reader Infinite 200 PRO series; Tecan Trading AG, Männedorf, Switzerland) with excitation and emission wavelengths of 270 nm and 545 nm, respectively. To allow comparability between samples, the DPA concentration was calculated in mmol per cell.

HP treatment, isolation of SD spores, and determination of DPA content were repeated three times on different days. The results are expressed as the mean of the replicates ± standard deviation.

### Proteome analysis.

A comparative quantitative protein analysis was conducted between the dormant and SD spores to identify proteins potentially involved in mHP germination. SD spores were isolated from the same spore batch in four independent isolations as described in “Isolation of superdormant spores,” after a 4-min pressure dwell time at 150 MPa.

### (i) Sample preparation for proteome analysis.

Samples were processed using a commercial iST kit (PreOmics, Planegg/Martinsried, Germany) with an updated version of the protocol. The initial dormant spores and isolated SD spores (four samples per category) were adjusted to an approximate value concentration of 10^8^ spores/mL using a Neubauer counting chamber. Approximately 400 μL of this suspension was pelleted and resuspended in 100 μL PreOmics lyse buffer. Spores in the lyse buffer were treated with 0.1-mm zirconium-silica beads in a bead mill homogenizer at 6.8 m/s (Omni Bead Ruptor 24 attached to an Omni Cryo cooling unit; Biovendis Products, Mannheim, Germany) to disrupt the spores and release the proteins. Seven cycles of 20 s each with a 60-s pause between each cycle were performed. Protein degradation during bead milling was avoided by cooling the chamber with liquid nitrogen. Successful cell disruption was verified using a microscope (DM6; Leica Microsystems, Wetzlar, Germany). It is worth noting that some of the spore’s coat proteins may not be extracted and may be retained in the insoluble fraction, and hence, they will not be detected in later analysis (39). The cell pellets solubilized in lyse buffer were then boiled at 95°C for 10 min and processed with high intensity focused ultrasound (HIFU) (Hielscher, Teltow, Germany) for 30 s with an ultrasonic amplitude of 85%. The samples were digested by adding 10 μL of the “digest” solution. After 60 min of incubation at 37°C, the digestion was interrupted with 100 μL of the “stop” solution. The solution was transferred to the cartridge, and the supernatant was removed using centrifugation at 3,800 × *g*, while the iST filter retained the peptides. Afterward, the peptides were washed, eluted, dried, and resolubilized in 20 μL of 3% acetonitrile and 0.1% formic acid. The peptide levels were normalized using the DS-11 series spectrophotometer (DeNovix, Inc., Wilmington, USA).

### (ii) LC-MS analysis.

Mass spectrometry analysis was performed on an Orbitrap Fusion Lumos instrument (Thermo Scientific, San Jose, USA) equipped with a digital PicoView source (New Objective, Littleton, USA) coupled to an M-Class ultraperformance liquid chromatography (UPLC) system (Waters GmbH, Wilmslow, UK). A solvent composition of 0.1% formic acid for channel A and 99.9% acetonitrile for channel B was used at the two channels. A total of 2 μL of peptides of each sample was loaded on a commercial MZ Symmetry C_18_ trap column (100 Å, 5 μm; 180 μm by 20 mm; Waters) followed by nanoEase MZ C_18_ HSS T3 column (100 Å, 1.8 μm; 75 μm by 250 mm; Waters). A flow rate of 300 nL/min with a gradient from 5% to 22% B in 80 min and 32% B in 10 min after an initial hold at 5% B for 3 min was used to elute the peptides. The column was washed with 95% B for 10 min, and then the column was re-equilibrated to the starting conditions for an additional 10 min. The samples were acquired in randomized order. The mass spectrometer was operated in data-dependent mode (DDA) to acquire full-scan MS spectra (300 to 1,500 *m/z*) at a resolution of 120,000 at 200 *m/z* after accumulation to a target value of 500,000. Data-dependent tandem MS (MS/MS) was recorded in the linear ion trap using quadrupole isolation with a window of 0.8 Da and higher-energy collisional dissociation (HCD) fragmentation with 35% fragmentation energy. Rapid scan mode was used to operate the ion trap with a target value of 10,000 and a maximum injection time of 50 ms. Precursors with an intensity of >5,000 were selected for MS/MS, and the maximum cycle time was set to 3 s. Charge state screening was enabled. Singly, unassigned and charge states of >7 were rejected. Precursor masses previously selected for MS/MS measurement were excluded from further selection for 20 s, and the exclusion window was set at 10 ppm. Internal lock mass calibration at *m/z* 371.1012 and 445.1200 was used to acquire the samples. A local laboratory information management system (62) was used to handle the mass spectrometry proteomics data. All relevant data were deposited to the ProteomeXchange Consortium via the PRIDE (http://www.ebi.ac.uk/pride) partner repository and can be viewed with the data set identifier PXD027768.

### (iii) Protein identification, label-free protein quantification, and statistical analysis.

MaxQuant (version 1.6.2.3) was used to process the acquired raw MS data, and the integrated Andromeda search engine (63) allowed protein identification. Spectra were searched against a B. subtilis strain 168 reference proteome (version 2020-08-31), concatenated to its reversed decoyed fasta database and common protein contaminants. N-terminal protein acetylation and methionine oxidation were set as variables, while carbamidomethylation of cysteine was set as a fixed modification. Enzyme specificity was set to trypsin/P, allowing a minimal peptide length of 7 amino acids and a maximum of 2 missed cleavages. Default settings of the MaxQuant Orbitrap were used. The maximum false discovery rate (FDR) was set to 0.05 for proteins and 0.01 for peptides. Label-free quantification was enabled, and a 2-min window for matching between runs was used. Each file was kept separate in the experimental design of the MaxQuant experimental design template to obtain individual quantitative values. Based on the intensity values reported in the proteinGroups.txt file, protein fold changes were computed. To filter for proteins with ≥2 peptides, allowing for a maximum of 4 missing values, and to normalize the data with a robust *Z*-score transformation to calculate *P* values using the *t* test with pooled variance, a set of functions implemented in the R package SRMService (64) was used. A pseudo fold change was computed for proteins where all measurements were missing in one of the conditions by replacing the missing group average with the mean of 10% of the smallest protein intensities in that condition. To allow a visualization of these proteins in a volcano plot, their −log_10_ (adjusted [adj.] *P* value) were set arbitrarily to the maximum *y* axis value of the mapped plot.

### (iv) Selection of proteins of interest based on proteome analysis.

Proteins that showed a log_2_ fold change [log_2_(FC)] of ≥|1| and an adjusted *P* value of <0.05 were defined as significantly differentially expressed between dormant and SD spores. These significantly differentially expressed proteins were screened for their putative function in the B. subtilis strain 168 database from the UniProt consortium (November 2020), and proteins associated with either germination or sporulation (which is tightly connected with germination) were selected as potential candidates of interest.

### Validation of protein roles in mHP germination by deletion mutants.

The roles of the selected proteins of interest in mHP germination were validated independently by comparing the germination capacity of B. subtilis deletion mutants lacking specific proteins with an otherwise isogenic wild-type strain 168. Deletion mutants and wild-type spores were sporulated in parallel as described in “Bacillus subtilis strains and spore preparation” and adjusted to a spore concentration of approximately 5 × 10^8^ spores/mL in 50 mM ACES (pH 7). Mutant spores and wild-type spores were HP treated at 150 MPa and 37°C for 3 min, as described in “HP-triggered spore germination.” A pressure dwell time of 3 min was chosen as this is the time after which the wild type showed approximately 10% SD spores. Hence, there is room to quantify both a slower and a possibly faster germination of the mutants. After the HP treatment, samples were put on ice and analyzed using FCM to determine germination, as described in “Determination of (super)dormant and germinated spore fraction.” To make the germination capacity of mutant strains and wild type directly comparable, the factor by which germination was reduced in the mutant strains was computed based on the ratio of the % of SD spores in the wild type to the mutant strains. This experiment was conducted with spores from three independent sporulation batches, whereas each mutant strain was sporulated at least twice independently from each other. For each mutant strain, experiments were repeated with two sporulation batches, and for each sporulation batch, HP treatments and FCM analyses were conducted at least in triplicate on different days.

### Data availability.

The proteomic data used in this study are available from the ProteomeXchange Consortium via the PRIDE (http://www.ebi.ac.uk/pride) partner repository with the data set identifier PXD027768.

## References

[1] Leggett MJ, Mcdonnell G, Denyer SP, Setlow P, Maillard JY. 2012. Bacterial spore structures and their protective role in biocide resistance. J Appl Microbiol 113:485–498. 10.1111/j.1365-2672.2012.05336.x. 22574673

[2] Wells-Bennik MHJ, Eijlander RT, den Besten HMW, Berendsen EM, Warda AK, Krawczyk AO, Nierop Groot MN, Xiao Y, Zwietering MH, Kuipers OP, Abee T. 2016. Bacterial spores in food: survival, emergence, and outgrowth. Annu Rev Food Sci Technol 7:457–482. 10.1146/annurev-food-041715-033144. 26934174

[3] Armenante PM, Akiti O. 2019. Sterilization processes in the pharmaceutical industry, p 311–379. *In* Ende MT, Ende DJ (ed), Chemical engineering in the pharmaceutical industry. John Wiley & Sons, Ltd, Hoboken, NJ.

[4] Reineke K, Mathys A. 2020. Endospore inactivation by emerging technologies: a review of target structures and inactivation mechanisms. Annu Rev Food Sci Technol 11:255–274. 10.1146/annurev-food-032519-051632. 31905011

[5] Lovdal IS, Hovda MB, Granum PE, Rosnes JT. 2011. Promoting *Bacillus cereus* spore germination for subsequent inactivation by mild heat treatment. J Food Prot 74:2079–2089. 10.4315/0362-028X.JFP-11-292. 22186048

[6] Zhang Y, Mathys A. 2018. Superdormant spores as a hurdle for gentle germination-inactivation based spore control strategies. Front Microbiol 9:3163. 10.3389/fmicb.2018.03163. 30662433 PMC6328458

[7] Zhang Y, Delbrück AI, Off CL, Benke S, Mathys A. 2019. Flow cytometry combined with single cell sorting to study heterogeneous germination of *Bacillus* spores under high pressure. Front Microbiol 10:3118. 10.3389/fmicb.2019.03118. 32038559 PMC6985370

[8] Setlow P, Wang S, Li Y-Q. 2017. Germination of spores of the orders *Bacillales* and *Clostridiales*. Annu Rev Microbiol 71:459–477. 10.1146/annurev-micro-090816-093558. 28697670

[9] Wuytack EY, Boven S, Michiels CW. 1998. Comparative study of pressure-induced germination of *Bacillus subtilis* spores at low and high pressures. Appl Environ Microbiol 64:3220–3224. 10.1128/AEM.64.9.3220-3224.1998. 9726863 PMC106713

[10] Paidhungat M, Setlow B, Daniels WB, Hoover D, Papafragkou E, Setlow P. 2002. Mechanisms of induction of germination of *Bacillus subtilis* spores by high pressure. Appl Environ Microbiol 68:3172–3175. 10.1128/AEM.68.6.3172-3175.2002. 12039788 PMC123951

[11] Black EP, Koziol-Dube K, Guan D, Wei J, Setlow B, Cortezzo DE, Hoover DG, Setlow P. 2005. Factors influencing germination of *Bacillus subtilis* spores via activation of nutrient receptors by high pressure. Appl Environ Microbiol 71:5879–5887. 10.1128/AEM.71.10.5879-5887.2005. 16204500 PMC1265928

[12] Black EP, Wei J, Atluri S, Cortezzo DE, Koziol-Dube K, Hoover DG, Setlow P. 2007. Analysis of factors influencing the rate of germination of spores of *Bacillus subtilis* by very high pressure. J Appl Microbiol 102:65–76. 10.1111/j.1365-2672.2006.03062.x. 17184321

[13] Reineke K, Mathys A, Heinz V, Knorr D. 2013. Mechanisms of endospore inactivation under high pressure. Trends Microbiol 21:296–304. 10.1016/j.tim.2013.03.001. 23540831

[14] Delbrück AI, Zhang Y, Heydenreich R, Mathys A. 2021. *Bacillus* spore germination at moderate high pressure: a review on underlying mechanisms, influencing factors, and its comparison with nutrient germination. Compr Rev Food Sci Food Saf 20:4159–4181. 10.1111/1541-4337.12789. 34147040

[15] Diehl P, Schauwecker J, Mittelmeier W, Schmitt M. 2008. High hydrostatic pressure, a novel approach in orthopedic surgical oncology to disinfect bone, tendons and cartilage. Anticancer Res 28:3877–3883. 19192644

[16] Chen D, Huang SS, Li YQ. 2006. Real-time detection of kinetic germination and heterogeneity of single *Bacillus* spores by laser tweezers Raman spectroscopy. Anal Chem 78:6936–6941. 10.1021/ac061090e. 17007517

[17] Stringer SC, Webb MD, Peck MW. 2011. Lag time variability in individual spores of *Clostridium botulinum*. Food Microbiol 28:228–235. 10.1016/j.fm.2010.03.003. 21315978

[18] Zhang P, Garner W, Yi X, Yu J, Li YQ, Setlow P. 2010. Factors affecting variability in time between addition of nutrient germinants and rapid dipicolinic acid release during germination of spores of *Bacillus* species. J Bacteriol 192:3608–3619. 10.1128/JB.00345-10. 20472791 PMC2897343

[19] Kong L, Doona CJ, Setlow P, Li Y. 2014. Monitoring rates and heterogeneity of high-pressure germination of *Bacillus* spores by phase-contrast microscopy of individual spores. Appl Environ Microbiol 80:345–353. 10.1128/AEM.03043-13. 24162576 PMC3911021

[20] Delbrück AI, Zhang Y, Hug V, Trunet C, Mathys A. 2021. Isolation, stability, and characteristics of high-pressure superdormant *Bacillus subtilis* spores. Int J Food Microbiol 343:109088. 10.1016/j.ijfoodmicro.2021.109088. 33621831

[21] Ghosh S, Scotland M, Setlow P. 2012. Levels of germination proteins in dormant and superdormant spores of *Bacillus subtilis*. J Bacteriol 194:2221–2227. 10.1128/JB.00151-12. 22343299 PMC3347068

[22] Wei J, Shah IM, Ghosh S, Dworkin J, Hoover DG, Setlow P. 2010. Superdormant spores of *Bacillus* species germinate normally with high pressure, peptidoglycan fragments, and bryostatin. J Bacteriol 192:1455–1458. 10.1128/JB.01497-09. 20047906 PMC2820858

[23] Ghosh S, Zhang P, Li YQ, Setlow P. 2009. Superdormant spores of *Bacillus* species have elevated wet-heat resistance and temperature requirements for heat activation. J Bacteriol 191:5584–5591. 10.1128/JB.00736-09. 19592590 PMC2737942

[24] Ghosh S, Setlow P. 2009. Isolation and characterization of superdormant spores of *Bacillus* species. J Bacteriol 191:1787–1797. 10.1128/JB.01668-08. 19136594 PMC2648361

[25] Zhang P, Kong L, Wang G, Scotland M, Ghosh S, Setlow B, Setlow P, Li Y-Q. 2012. Analysis of the slow germination of multiple individual superdormant *Bacillus subtilis* spores using multifocus Raman microspectroscopy and differential interference contrast microscopy. J Appl Microbiol 112:526–536. 10.1111/j.1365-2672.2011.05230.x. 22212253

[26] Chen Y, Ray WK, Helm RF, Melville SB, Popham DL. 2014. Levels of germination proteins in *Bacillus subtilis* dormant, superdormant, and germinating spores. PLoS One 9:e95781. 10.1371/journal.pone.0095781. 24752279 PMC3994143

[27] Ghosh S, Setlow P. 2010. The preparation, germination properties and stability of superdormant spores of *Bacillus cereus*. J Appl Microbiol 108:582–590. 10.1111/j.1365-2672.2009.04442.x. 19674187

[28] Griffiths KK, Zhang J, Cowan AE, Yu J, Setlow P. 2011. Germination proteins in the inner membrane of dormant *Bacillus subtilis* spores colocalize in a discrete cluster. Mol Microbiol 81:1061–1077. 10.1111/j.1365-2958.2011.07753.x. 21696470 PMC7959159

[29] Li Y, Jin K, Ghosh S, Devarakonda P, Carlson K, Davis A, Stewart KV, Cammett E, Rossi PP, Setlow B, Lu M, Setlow P, Hao B. 2014. Structural and functional analysis of the GerD spore germination protein of *Bacillus* species. J Mol Biol 426:1995–2008. 10.1016/j.jmb.2014.02.004. 24530795 PMC4038265

[30] Luu S, Cruz-Mora J, Setlow B, Feeherry FE, Doona CJ, Setlow P. 2015. The effects of heat activation on *Bacillus* spore germination, with nutrients or under high pressure, with or without various germination proteins. Appl Environ Microbiol 81:2927–2938. 10.1128/AEM.00193-15. 25681191 PMC4375313

[31] Setlow P. 2014. Germination of spores of *Bacillus* species: what we know and do not know. J Bacteriol 196:1297–1305. 10.1128/JB.01455-13. 24488313 PMC3993344

[32] Paidhungat M, Setlow B, Driks A, Setlow P. 2000. Characterization of spores of *Bacillus subtilis* which lack dipicolinic acid. J Bacteriol 182:5505–5512. 10.1128/JB.182.19.5505-5512.2000. 10986255 PMC110995

[33] Nguyen Thi Minh H, Guyot S, Perrier-Cornet J-M, Gervais P. 2008. Effect of the osmotic conditions during sporulation on the subsequent resistance of bacterial spores. Appl Microbiol Biotechnol 80:107–114. 10.1007/s00253-008-1519-x. 18506440

[34] Melly E, Genest PC, Gilmore ME, Little S, Popham DL, Driks A, Setlow P. 2002. Analysis of the properties of spores of *Bacillus subtilis* prepared at different temperatures. J Appl Microbiol 92:1105–1115. 10.1046/j.1365-2672.2002.01644.x. 12010551

[35] Magge A, Granger AC, Wahome PG, Setlow B, Vepachedu VR, Loshon CA, Peng L, Chen D, Li YQ, Setlow P. 2008. Role of dipicolinic acid in the germination, stability, and viability of spores of *Bacillus subtilis*. J Bacteriol 190:4798–4807. 10.1128/JB.00477-08. 18469099 PMC2446992

[36] Kort R, O'Brien AC, van Stokkum IHM, Oomes SJCM, Crielaard W, Hellingwerf KJ, Brul S. 2005. Assessment of heat resistance of bacterial spores from food product isolates by fluorescence monitoring of dipicolinic acid release. Appl Environ Microbiol 71:3556–3564. 10.1128/AEM.71.7.3556-3564.2005. 16000762 PMC1169001

[37] Hindle AA, Hall EAH. 1999. Dipicolinic acid (DPA) assay revisited and appraised for spore detection. Analyst 124:1599–1604. 10.1039/a906846e. 10746319

[38] Li Z, Schottroff F, Simpson DJ, Gänzle MG. 2019. The copy number of the spoVA2mob operon determines pressure resistance of *Bacillus* endospores. Appl Environ Microbiol 85:e01596-19. 10.1128/AEM.01596-19. 31375487 PMC6752002

[39] Swarge BN, Roseboom W, Zheng L, Abhyankar WR, Brul S, de Koster CG, de Koning LJ. 2018. “One-Pot” sample processing method for proteome-wide analysis of microbial cells and spores. Prot Clin Appl 12:1700169. 10.1002/prca.201700169. PMC6174930 29484825

[40] Fan L, Hou F, Idris Muhammad A, Bilyaminu Ismail B, Lv R, Ding T, Liu D. 2020. Proteomic responses of spores of *Bacillus subtilis* to thermosonication involve large-scale alterations in metabolic pathways. Ultrason Sonochem 64:104992. 10.1016/j.ultsonch.2020.104992. 32018137

[41] Doona CJ, Ghosh S, Feeherry FF, Ramirez-Peralta A, Huang Y, Chen H, Setlow P. 2014. High pressure germination of *Bacillus subtilis* spores with alterations in levels and types of germination proteins. J Appl Microbiol 117:711–720. 10.1111/jam.12557. 24891141

[42] Pelczar PL, Igarashi T, Setlow B, Setlow P. 2007. Role of GerD in germination of *Bacillus subtilis* spores. J Bacteriol 189:1090–1098. 10.1128/JB.01606-06. 17122337 PMC1797312

[43] Jamroškovič J, Pavlendová N, Muchová K, Wilkinson AJ, Barák I. 2012. An oscillating Min system in *Bacillus subtilis* influences asymmetrical septation during sporulation. Microbiology (Reading) 158:1972–1981. 10.1099/mic.0.059295-0. 22628484 PMC3542138

[44] Barák I, Muchová K, Labajová N. 2019. Asymmetric cell division during *Bacillus subtilis* sporulation. Future Microbiol 14:353–363. 10.2217/fmb-2018-0338. 30855188

[45] Barák I, Muchová K. 2018. The positioning of the asymmetric septum during sporulation in *Bacillus subtilis*. PLoS One 13:e0201979. 10.1371/journal.pone.0201979. 30092000 PMC6084994

[46] Bagyan I, Noback M, Bron S, Paidhungat M, Setlow P. 1998. Characterization of yhcN, a new forespore-specific gene of *Bacillus subtilis*. Gene 212:179–188. 10.1016/s0378-1119(98)00172-3. 9611260

[47] Zheng L, Abhyankar W, Ouwerling N, Dekker HL, Van Veen H, Van Der Wel NN, Roseboom W, De Koning LJ, Brul S, De Koster CG. 2016. *Bacillus subtilis* spore inner membrane proteome. J Proteome Res 15:585–594. 10.1021/acs.jproteome.5b00976. 26731423

[48] Kuwana R, Kasahara Y, Fujibayashi M, Takamatsu H, Ogasawara N, Watabe K. 2002. Proteomics characterization of novel spore proteins of *Bacillus subtilis*. Microbiology (Reading) 148:3971–3982. 10.1099/00221287-148-12-3971. 12480901

[49] Johnson CL, Moir A. 2017. Proteins YlaJ and YhcN contribute to the efficiency of spore germination in *Bacillus subtilis*. FEMS Microbiol Lett 364:fnx047. 10.1093/femsle/fnx047. 28333204

[50] Luu S, Setlow P. 2014. Analysis of the loss in heat and acid resistance during germination of spores of *Bacillus* species. J Bacteriol 196:1733–1740. 10.1128/JB.01555-14. 24563034 PMC3993331

[51] Henriques AO, Bryan EM, Beall BW, Moran CP. 1997. cse15, cse60, and csk22 are new members of mother-cell-specific sporulation regulons in *Bacillus subtilis*. J Bacteriol 179:389–398. 10.1128/jb.179.2.389-398.1997. 8990290 PMC178708

[52] Cabrera-Hernandez A, Setlow P. 2000. Analysis of the regulation and function of five genes encoding small, acid-soluble spore proteins of *Bacillus subtilis*. Gene 248:169–181. 10.1016/s0378-1119(00)00125-6. 10806362

[53] Galperin MY, Mekhedov SL, Puigbo P, Smirnov S, Wolf YI, Rigden DJ. 2012. Genomic determinants of sporulation in Bacilli and Clostridia: towards the minimal set of sporulation-specific genes. Environ Microbiol 14:2870–2890. 10.1111/j.1462-2920.2012.02841.x. 22882546 PMC3533761

[54] Korza G, Camilleri E, Green J, Robinson J, Nagler K, Moeller R, Caimano MJ, Setlow P. 2019. Analysis of the mRNAs in spores of *Bacillus subtilis*. J Bacteriol 201:e00007-19. 10.1128/JB.00007-19. 30782632 PMC6456856

[55] Swarge B, Abhyankar W, Jonker M, Hoefsloot H, Kramer G, Setlow P, Brul S, de Koning LJ. 2020. Integrative analysis of proteome and transcriptome dynamics during *Bacillus subtilis* spore revival. mSphere 5:e00463-20. 10.1128/mSphere.00463-20. 32759332 PMC7407066

[56] Paidhungat M, Setlow P. 2000. Role of ger proteins in nutrient and nonnutrient triggering of spore germination in *Bacillus subtilis*. J Bacteriol 182:2513–2519. 10.1128/JB.182.9.2513-2519.2000. 10762253 PMC111315

[57] Koo BM, Kritikos G, Farelli JD, Todor H, Tong K, Kimsey H, Wapinski I, Galardini M, Cabal A, Peters JM, Hachmann AB, Rudner DZ, Allen KN, Typas A, Gross CA. 2017. Construction and analysis of two genome-scale deletion libraries for *Bacillus subtilis*. Cell Syst 4:291–305. 10.1016/j.cels.2016.12.013. 28189581 PMC5400513

[58] Hashimoto T, Frieben WR, Conti SF. 1969. Germination of single bacterial spores. J Bacteriol 98:1011–1020. 10.1128/jb.98.3.1011-1020.1969. 4977979 PMC315288

[59] Kong L, Zhang P, Wang G, Yu J, Setlow P, Li YQ. 2011. Characterization of bacterial spore germination using phase-contrast and fluorescence microscopy, Raman spectroscopy and optical tweezers. Nat Protoc 6:625–639. 10.1038/nprot.2011.307. 21527920

[60] Lindsay JA, Beaman TC, Gerhardt P. 1985. Protoplast water content of bacterial spores determined by buoyant density sedimentation. J Bacteriol 163:735–737. 10.1128/jb.163.2.735-737.1985. 4019413 PMC219183

[61] Rosen DL, Sharpless C, McGown LB. 1997. Bacterial spore detection and determination by use of terbium dipicolinate photoluminescence. Anal Chem 69:1082–1085. 10.1021/ac960939w.

[62] Türker C, Akal F, Joho D, Panse C, Barkow-Oesterreicher S, Rehrauer H, Schlapbach R. 2010. B-fabric: the Swiss army knife for life sciences, p 717–720. *In* Advances in Database Technology—EDBT 2010. 13th International Conference on Extending Database Technology, Proceedings, Lausanne, Switzerland.

[63] Cox J, Mann M. 2008. MaxQuant enables high peptide identification rates, individualized p.p.b.-range mass accuracies and proteome-wide protein quantification. Nat Biotechnol 26:1367–1372. 10.1038/nbt.1511. 19029910

[64] Wolski W, Grossmann J, Panse C. 2018. SRMService - R-package to report quantitative mass spectrometry data. http://github.com/protViz/SRMService.

[65] Setlow B, Setlow P. 1996. Role of DNA repair in *Bacillus subtilis* spore resistance. J Bacteriol 178:3486–3495. 10.1128/jb.178.12.3486-3495.1996. 8655545 PMC178117

[66] Kunst F, Ogasawara N, Moszer I, Albertini AM, Alloni G, Azevedo V, Bertero MG, Bessières P, Bolotin A, Borchert S, Borriss R, Boursier L, Brans A, Braun M, Brignell SC, Bron S, Brouillet S, Bruschi CV, Caldwell B, Capuano V, Carter NM, Choi SK, Codani JJ, Connerton IF, Cummings NJ, Daniel RA, Denizot F, Devine KM, Düsterhöft A, Ehrlich SD, Emmerson PT, Entian KD, Errington J, Fabret C, Ferrari E, Foulger D, Fritz C, Fujita M, Fujita Y, Fuma S, Galizzi A, Galleron N, Ghim SY, Glaser P, Goffeau A, Golightly EJ, Grandi G, Guiseppi G, Guy BJ, Haga K, Haiech J, et al. 1997. The complete genome sequence of the gram-positive bacterium *Bacillus subtilis*. Nature 390:249–256. 10.1038/36786. 9384377

