# Supplementary material for: Moderate High-Pressure Superdormancy in Bacillus Spores: Properties of Superdormant Spores and Proteins Potentially Influencing Moderate High-Pressure Germination
Source: Appl Environ Microbiol. 2022 Feb 22;88(4):e02406-21. doi: 10.1128/aem.02406-21 (PMC8863042; doi:10.1128/aem.02406-21)
Supplement: Supplemental file 1 — Fig. S1. Download aem.02406-21-s0001.pdf, PDF file, 0.2 MB [file aem.02406-21-s0001.pdf]

Supplementary Material

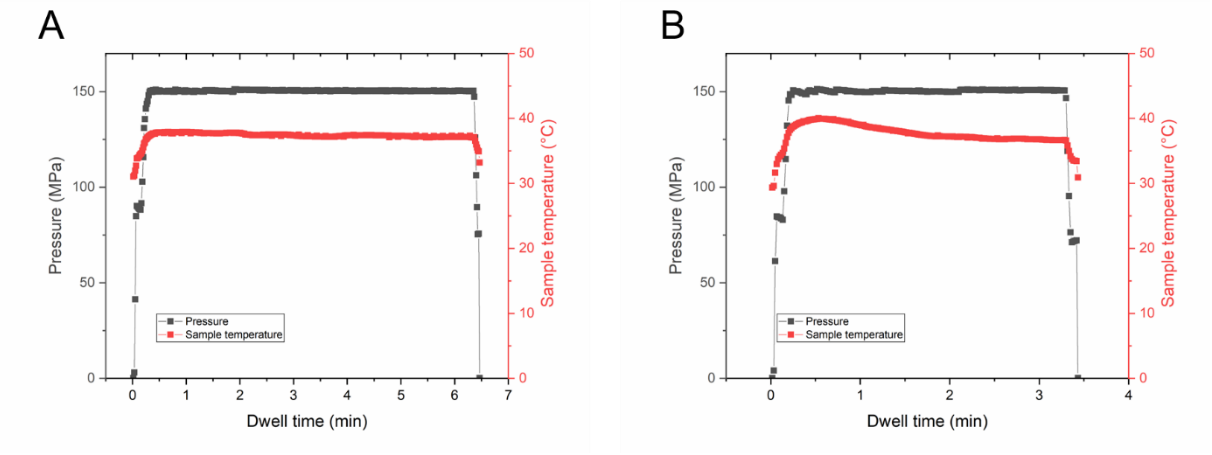

Supplementary Figure S1: Representative p/T/t profiles for high pressure treated *Bacillus subtilis* spores at 150 MPa and 37°C. A) Treatment in 1.5 mL cryotube vials for 6 min, B) treatment in 200 µL polyethylene pouches for 3 min.
